# Neurodegeneration Caused by S1P-Lyase Deficiency Involves Calcium-Dependent Tau Pathology and Abnormal Histone Acetylation

**DOI:** 10.3390/cells9102189

**Published:** 2020-09-28

**Authors:** Shah Alam, Antonia Piazzesi, Mariam Abd El Fatah, Maren Raucamp, Gerhild van Echten-Deckert

**Affiliations:** 1 LIMES Institute for Membrane Biology and Lipid Biochemistry, Kekulé-Institute, University of Bonn, 53121 Bonn, Germany; shah.bonn@outlook.com (S.A.); piazzesa@hushmail.com (A.P.); mariam@uni-bonn.de (M.A.E.F.); 2 Institute of Physiology, University Bonn, 53111 Bonn, Germany; Maren.Raucamp@ukb.uni-bonn.de

**Keywords:** Sphingosine 1-phosphate (S1P), S1P-lyase (SGPL1), tau, calcium, histone acetylation, hippocampus, cortex, astrocytes, neurons

## Abstract

We have shown that sphingosine 1-phosphate (S1P) generated by sphingosine kinase 2 (SK2) is toxic in neurons lacking S1P-lyase (SGPL1), the enzyme that catalyzes its irreversible cleavage. Interestingly, patients harboring mutations in the gene encoding this enzyme (*SGPL1*) often present with neurological pathologies. Studies in a mouse model with a developmental neural-specific ablation of SGPL1 (SGPL1^fl/fl/Nes^) confirmed the importance of S1P metabolism for the presynaptic architecture and neuronal autophagy, known to be essential for brain health. We now investigated in SGPL1-deficient murine brains two other factors involved in neurodegenerative processes, namely tau phosphorylation and histone acetylation. In hippocampal and cortical slices SGPL1 deficiency and hence S1P accumulation are accompanied by hyperphosphorylation of tau and an elevated acetylation of histone3 (H3) and histone4 (H4). Calcium chelation with BAPTA-AM rescued both tau hyperphosphorylation and histone acetylation, designating calcium as an essential mediator of these (patho)physiological functions of S1P in the brain. Studies in primary cultured neurons and astrocytes derived from SGPL1^fl/fl/Nes^ mice revealed hyperphosphorylated tau only in SGPL1-deficient neurons and increased histone acetylation only in SGPL1-deficient astrocytes. Both could be reversed to control values with BAPTA-AM, indicating the close interdependence of S1P metabolism, calcium homeostasis, and brain health.

## 1. Introduction

Sphingosine 1-phosphate (S1P), an evolutionarily conserved catabolic intermediate of sphingolipid metabolism, regulates diverse biological processes in the brain including neural development, differentiation, and survival [1,2]. S1P exerts its functions either as a ligand of five specific G-protein coupled receptors (S1PR1-5) or alternatively as an intracellular second messenger [3,4]. Notably, one of the first described intracellular, receptor-independent effects of S1P is its involvement in calcium homeostasis [5,6]. 

S1P-lyase (SGPL1) irreversibly cleaves S1P in the final step of sphingolipid catabolism, generating ethanolamine phosphate and a long-chain aldehyde [7]. Of interest, in 2017 different research groups reported patients and their relatives harboring autosomal recessive mutations in *SGPL1* and exhibiting a variety of pathologies including congenital steroid-resistant nephrotic syndrome, primary adrenal insufficiency, and last but not least central and peripheral neurological defects [8]. 

The essential role of S1P in brain development became clear years ago, when elimination of S1P production was shown to severely disturb neurogenesis including neural tube closure, and angiogenesis leading to embryonic death [9]. Yet, reports on the involvement of S1P in the pathology of neurodegenerative diseases including Alzheimer’s disease (AD) are rather conflicting [10]. On the one hand, it is assumed that loss of the neuroprotective factor S1P occurs early in AD pathogenesis [11]. Indeed reduced expression of sphingosine kinase 1, one of the two sphingosine kinases known to generate S1P, and a simultaneous augmented expression of SGPL1 were detected in AD brains [12]. On the other hand, S1P was shown to stimulate neuronal beta-site amyloid precursor protein (APP) cleaving enzyme (BACE1) that catalyzes the rate-limiting step of the formation of amyloid beta peptide (Aβ), the major component of senile plaques in AD [13]. In addition, increased S1P levels were shown to induce death of terminally differentiated post-mitotic neurons [14,15]. Moreover, S1P was found to be increased in cerebrospinal fluid during early stages of AD [16].

In an attempt to clarify the function of S1P in the brain, we generated a mouse model in which SGPL1 was inactivated specifically in neural cells (SGPL1^fl/fl/Nes^). As expected, SGPL1 ablation leads to S1P accumulation in the brain which was found to affect presynaptic architecture and function [17]. In addition, we demonstrated that SGPL1 deficiency blocks neuronal autophagy at its early stages because of reduced phosphatidylethanolamine (PE) production [18]. Consequently, an accumulation of aggregate-prone proteins such as APP and α-synuclein (SNCA) was detected. All these molecular changes in neurons were accompanied by deficits in motor coordination as well as in spatial and associative learning and memory [17,18].

We have also shown that S1P promotes excessive phosphorylation of tau in neurons generated from SGPL1 systemic knockout mice [14]. Note that tau is the major neuronal microtubule assembly activator protein and there is no doubt regarding its essential involvement in the etiopathogenesis of AD and a family of related neurodegenerative disorders known as tauopathies [19]. Tau neurotoxicity has been linked to heterochromatin relaxation and hence to aberrant gene expression in tauopathies [20]. Studies in primary cultured neurons revealed that nuclear tau directly regulates pericentromericheterochtomatin integrity that appears disrupted in AD neurons [21]. Recently, an epigenome-wide study in which acetylation of lysine 9 of histone3 (H3K9ac) was used as a marker for transcriptionally active open chromatin, also led to the conclusion that in aging and AD brains tau pathology drives chromatin rearrangement [22]. Furthermore, in post-mortem AD brains, increased levels of acetylated H3 and H4 were detected and correlated with the load of hyperphosphorylated tau [23].

Remarkably, in a tumorigenic cell line, S1P generated by sphingosine kinase 2 (SK2) was reported to specifically enhance acetylation of H3 and H4 at K9 and K5, respectively, by directly inhibiting histone deacetylases 1 and 2 (HDACs 1, 2) [24]. In the present study, we demonstrate that accumulation of S1P as a result of SGPL1 deficiency increases tau phosphorylation and histone acetylation also in brain slices. Furthermore, we found that both effects can be rescued in the presence of the calcium chelator BAPTA-AM, indicating that this process is calcium-dependent. Notably, these effects were cell-type specific, with increases in tau phosphorylation and histone acetylation found in neurons and astrocytes, respectively. Taken together, our results further elucidate the extensive and complex interrelation of S1P metabolism and brain health. 

## 2. Materials and Methods

### 2.1. List of Abbreviations

AD: Alzheimer’s disease; APP: amyloid precursor protein; ac: acetylated; H3: histone3; H4: histone4; H2B, histone2B; K5, lysine residue 5; K9, lysine residue 9; K12, lysine residue 12; HDAC: histone deacetylase; S1P: sphingosine 1-phosphate; SGPL1: S1P-lyase; SK: sphingosine kinase; S1PR: S1P receptor; S396/404, serine residue 396 and serine residue 404; S262/356, serine residue 262 and serine residue 356.

### 2.2. Antibodies and Chemicals

Monoclonal antibody against phosphorylated tau PHF1 (S396/404), 12E8(S262/356) and against total tau (K9JA) was a kind gift from Prof. Dr. Eckhard Mandelkow and Prof. Dr. Eva-Maria Mandelkow (DZNE, University of Bonn, Germany). Acetyl-Histone H3 antibody sampler kit comprising acetylation of K9, K14, K18, K27, and K56, anti-H4K5ac, and anti-H2BK12ac antibody were from Cell Signaling Technology Danvers, MA, USA (9927, 8647 and 5410). Anti-HDAC1, -HDAC2, -HDAC3, and -HDAC6 antibodies were from Cell Signaling Technology (antibody Sampler Kit #9928). Anti-SGPL1 antibody was from abcam (Cambridge, UK; ab56183) and Anti-glial fibrillary acidic protein (GFAP) antibody from Cell Signaling Technology (12389).Secondary antibodies were HRP linked anti-rabbit and anti-mouse IgG (Cell Signaling Technology, 7074 and 7076). 5,5′-Dimethyl-BAPTA-AM was from Sigma- Aldrich, Munich, Germany (16609).

### 2.3. Animals

The SGPL1^flox/flox^ lines were generated as recently described [17]. SGPL1^flox/flox^ mice, harboring “floxed” exons 10–12 on both Sgpl1 alleles were crossbred with mice expressing the Nes (nestin) - Cre transgene. Thus SGPL1^fl/fl/Nes^mice (nKO) in which “floxed” exons are excised by Cre recombinase were obtained. For all the experiments, the floxed mice (SGPL1^fl/fl^) served as controls. Brain tissue was taken from mice housed in standard conditions at the University of Bonn.

### 2.4. Ethical Statement

All animal experiments were conducted in accordance with the guidelines of the Animal Care Committee of the University of Bonn. The experimental protocols were approved by Landesamt für Natur, Umwelt und Verbraucherschutz (LANUV) Nordrhein-Westfalen (NRW) (LANUV NRW, Az. 81–02.05.40.19.013).

### 2.5. Cell Culture

Primary neuronal culture: Granular cells were cultured from the cerebella of 6-days old mice as described previously [25]. Briefly, neurons were isolated by mild trypsinization (0.05%, w/v; Sigma-Aldrich, Munich, Germany P6567) and dissociated by passing them repeatedly through a constricted Pasteur pipette in a DNase solution (0.1%, w/v; Roche, Basel, Switzerland 04716728001). The cells were then suspended in Dulbecco’s modified Eagle’s medium (Thermo Fisher Scientific, Waltham, MA, USA 10566032) containing 10% heat-inactivated horse serum (Thermo Fisher Scientific, 16050130) supplemented with 100 units/mL penicillin and 100 mg/mL streptomycin (Gibco™ Thermo Scientific 15140122) and plated onto precoated poly-L-lysine (Sigma-Aldrich, P6282) 6-well plates, 35 mm in diameter (Sarstedt, Nümbrecht, Germany (83.3920.300). Twenty-four h after plating, 1% cytosine ß-D-arabinofuranoside hydrochloride (Sigma-Aldrich, C6645) was added to the medium to arrest the division of non-neuronal cells. After 10 days in culture, cells were used for experiments as indicated.

Primary astrocyte culture: Mixed cortical cell isolation for astrocyte culture was performed using P1 to P4 mouse pups as described previously [26]. Briefly, cerebral cortices were dissected in Ca^2+^- and Mg^2+^-free HBSS (Gibco^TM^, Thermo Scientific, 14185652) and incubated in 0.125% trypsin for 10 min at 37 °C. The resulting cell suspension was diluted in complete media Dulbecco’s modified Eagle’s medium supplemented 10% fetal bovine serum (PAN biotech, Aidenbach, Germany, P40-47100) and 1% penicillin/ streptomycin. The cell suspension was plated on poly-L-Lysine (P-1399) coated T75 cell culture flasks and kept at 37 °C in a humidified 5% CO_2_ incubator. Medium was renewed every 2 days. After about 21 days, flasks were shaken horizontally, and the medium containing detached microglia and oligodendrocyte precursor cells (OPC) was removed. Later, astrocytes were collected and seeded onto 6-well cell culture dishes (35 mm diameter) and used for experiments after 24 h, as indicated.

### 2.6. BAPTA- AM Treatment

Hippocampal and cortical slices of 200 µm thickness were prepared in ice-cold high sucrose solution (220 mM sucrose, 26 mM NaHCO_3_, 10 mM glucose, 6 mM MgSO_4_.7H_2_O, 3 mM KCL solid, 1.25 mM NaH_2_PO_4_. H_2_O, 0.43 mM CaCl_2_) gassed with carbogen. Then, both hippocampal and cortical slices were incubated in artificial cerebrospinal fluid (119 mMNaCl, 26.2 mM NaHCO_3_, 2.5 mMKCl, 1 mM NaH_2_PO_4_, 1.3 mM MgCl_2_, 10 mM glucose) with and without 150 µM BAPTA-AM for 2 h and kept at −80 °C until use.

### 2.7. Western Immunoblotting

Tissue and cell samples were homogenized in RIPA buffer (20 mMTris-HCl, pH 7.5, 150 mMNaCl, 1 mM EDTA, 1 mM EGTA, 1% NP-40 (Thermo Fisher Scientific, Waltham, MA, USA, FNN0021), 1% Nadcap (Sigma-Aldrich, Munich, Germany, D6750), 2.5 mM Na_4_P_2_O_7_, 1 mM b-glycerophosphate, 1 mM Na_3_VO_4_, 1 mg/mL leupeptin (Thermo Fisher Scientific, 78435). Samples were kept on ice for 1 h followed by centrifugation at 14,000× rpm at 4 °C for 45 min. The protein concentration of the supernatants was determined using the BCA assay (Sigma–Aldrich). Samples were stored at −20 °C until use. Laemmli Sample Buffer (Bio-rad Laboratories, Munich, Germany, 1610747) was added to lysates and samples were heated for 10 min at 95 °C before loading on SDS-PAGE gel. Proteins were separated by SDS-PAGE in running buffer (25 mM Tris, pH 8.3, 192 mM glycine, 0.1% SDS) at 50 V for 15 min, then 1 h at 150 V. Transfer onto nitrocellulose membranes (Porablot NCL; Macherey-Nagel, Thermo Fisher Scientific, 741290) was performed at 4 °C and 400 mA for 2 h in blotting buffer (50 mMTris, pH 9.2, 40 mM glycine, 20% methanol). Membranes were blocked with 5% milk powder (Bio-Rad Laboratories, 1706404) in TBS-Tween 20 (20 mM Tris, pH 7.5, 150 mM NaCl, 0.1% Tween 20, Sigma-Aldrich, P9416) for 1 h, washed 3 times (10 min each) and incubated at 4 °C overnight with the primary antibody. Then membranes were washed again and incubated for 1 h at room temperature with an HRP-conjugated secondary antibody. Western BLoT Chemiluminescence HRP Substrate (TAKARA Bio, Saint-Germain-en-Laye, France, T7101B) was used for detection with the VersaDoc 5000 imaging system (Bio-Rad, Hercules, CA, USA). β-actin was used as loading control. Quantification was performed using ImageJ and Prism GraphPad program.

### 2.8. RNA Isolation and Real-Time PCR

Up to 1 µg of total RNA (isolated with EXTRAzol from Blirt, 7Bioscience, Hartheim/Rhein, Germany, EM30-200) was used for reverse transcription with the ProtoScript^®^ II First Strand cDNA Synthesis kit (New England Biolabs, Frankfurt/Main, Germany, E6560L). The resulting total cDNA was then applied to real-time PCR (CFX96-real time PCR, Bio-Rad Laboratories, Munich, Germany) using β-actin and 18S RNA as housekeeping genes. The primers for real-time PCR were designed using the online tool from NCBI BLAST primer and obtained from Invitrogen, Carlsbad, CA, USA. They are listed as follows: name: forward primer (for), reverse primer (rev): β-actin, 5′-CTTTGCAGCTCCTTCGTTGC (for) and 5′-CCTTCTGACCCATTCCCACC (rev); 18S RNA, 5′-CCCCTCGATGCTCTTAGCTG (for) and 5′-CTTTCGCTCTGGTCCGTCTT (rev); HDAC1, 5′-AGCTGGGCTTTCCAAGTTACC (for) and 5′-TGGTCCACACCCTTCTCGTA (rev); HDAC2, 5′-CGGCCAAGCCTGACTTAGAT (for) and 5′-TTTTCAGCTGTCCTCGGTGG (rev); HDAC3, 5′-TGCCCCAGATTTCACACTCC (for) and 5′-TGGTCCAGATACTGGCGTGA (rev); HDAC6, 5′- GGCGCAGATTAGAGAGCCTT (for) and 5′-GAAGGGGTGACTGGGGATTG (rev); SGPL1, 5′-TTTCCTCATGGTGTGATGGA (for) and 5′-C CCCAGACAAGCATCCAC3(rev).The reactions were performed at 95 °C for 30 s, 95 °C for 10 s, and 60 °C for 1 min. Relative normalized mRNA expression was obtained from real-time qPCR. Statistical significance of the relative normalized mRNA expression was determined by *t*-test in Prism GraphPad program.

### 2.9. Immunohistochemistry

Brains were removed and quick-frozen in liquid nitrogen. Cryo-sectioning was used to produce 10 µm sagittal sections, which were placed on Superfrost Plus positively charged microscope slides. Brain sections were fixed for 5 min in ice-cold 4% (*v*/*v*) paraformaldehyde in phosphate-buffered saline (PBS). Sections were then permeabilized with 0.1% (*v*/*v*) Triton X-100 in PBS for 30 min at room temperature (RT). Tissue sections were blocked in 20% (*v*/*v*) normal goat serum in PBS for 30 min and incubated overnight at 4 °C with primary antibody (PHF1 and 12E8). The primary antibodies were diluted 1:200 in PBS containing 0.5% lambda-carrageenan (Sigma Aldrich, Munich, Germany, 22049) and 0.02% sodium azide and applied overnight to the sections at 4 °C. Following a washing step, brain sections were incubated with Cy3-conjugated anti-rabbit antibody diluted 1:300 in PBS with the same additions as above for 1 h at RT. Finally, antibody-labeled brain sections were embedded in Fluoromount G medium with DAPI (Electron Microscopy Sciences, Hatfield, PA, USA for microscopic analysis (Zeiss Axioskop 2 epi-fluorescence microscope equipped with a digital Zeiss AxioCamHRc camera, Carl Zeiss Jena, Jena, Germany).

### 2.10. Immunocytochemistry

Cover slips with astrocytes were rinsed 3 times with PBS at room temperature (RT) and then fixed in methanol (−20 °C, 5 min). Between each incubation step cells were always rinsed 3 times with PBS. Then cells were blocked in 20% (*v*/*v*) normal goat serum in PBS for 30 min. and incubated overnight with anti-GFAP antibody diluted 1:200 with PBS at 4 °C and thenwith anti-rabbit Alexa Fluor 488 (1:300)-conjugated secondary antibodies (Invitrogen, Carlsbad, CA, USA) for 50 min at RT. Finally, cells were embedded in Fluoromount G medium with DAPI for microscopic analyses (Zeiss Axioskop 2 epi-fluorescence microscope equipped with a digital Zeiss AxioCamHRc camera, Carl Zeiss Jena, Jena, Germany).

### 2.11. Statistical Analysis

The GraphPad Prism 5 software was used for statistical analysis.All values are expressed as means ± SEMobtained from at least 3 independent experiments. The significanceof differences between the experimental groups and controls was assessed by either Student’s *t*-test with false discovery rate (FDR) correction or One-Way ANOVA, as appropriate. *p/q*< 0.05 was considered statistically significant (* *p/q* < 0.05; ** *p/q* < 0.01; *** *p/q* < 0.001; compared with the respective control group).

## 3. Results 

### 3.1. Elevated Phosphorylation of Tau in SGPL1-Deficient Brains Is Cell Type Specific 

**Figure 1:**
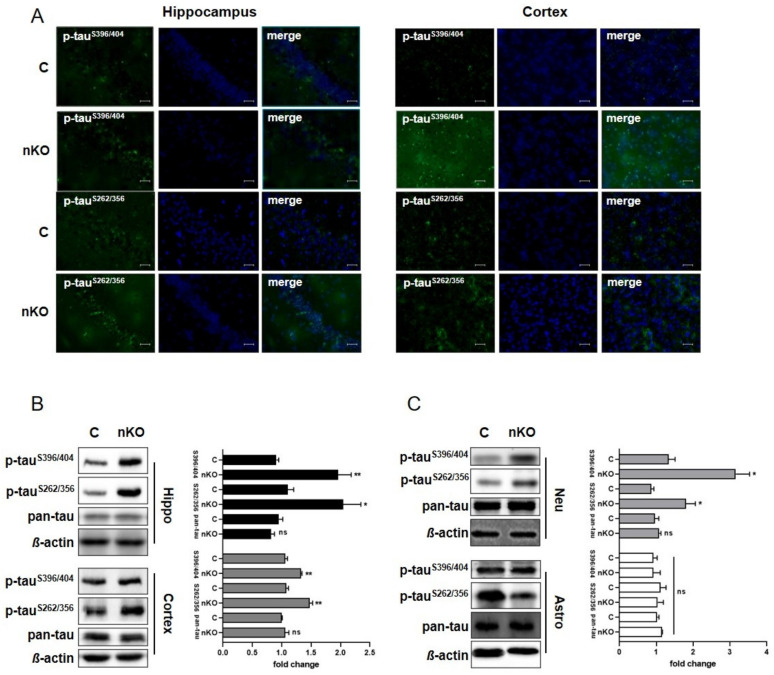
SGPL1 deficiency results in tau hyperphosphorylation in the brain. (**A**) Representative images of hippocampal and cortical slices stained for phospho-tau^S396/404^, phospho-tau^S262/356^, and DAPI from control (**C**) and SGPL1^fl/fl/Nes^ (nKO) mice. Scale bar: 200 µm. (**B**,**C**) Protein quantification of phospho-tau^S396/404^, phospho-tau^S262/356^, and total tau (pan-tau) in the hippocampus (Hippo, black), cortex (dark grey), neurons (Neu, light grey) and astrocytes (Astro, white) in control (**C**) and SGPL1^fl/fl/Nes^ (nKO) mice. Bars mean +/− S.E.M, Student’s *t*-test with false discovery rate (FDR) correction, n = 3–5, * *q* < 0.05, ** *q* < 0.01, ns = not significant.

We have previously shown that tau phosphorylation is elevated in primary cultured neurons derived from brains of systemic SGPL1-knockout (KO) mice [14]. Here, we generated a neural-specific Sgpl1 knockout (SGPL1^fl/fl/Nes^; nKO) mouse and performed our analysis in brain slices, primary neuronal and astrocyte cultures (Supplementary Figure S1). We have found that tau phosphorylation at disease-relevant sites is also significantly increased in hippocampal and cortical slices from SGPL1^fl/fl/Nes^mice consistent with our previously reported findings in systemic KO mice [14] (Figure 1A,B). Furthermore, this increase in tau phosphorylation at disease-relevant sites was not accompanied by changes in total tau levels (Figure 1B). A more refined analysis in primary cultured neurons and astrocytes from SGPL1^fl/fl/Nes^mice further revealed that this effect is primarily attributed to neurons, as tau phosphorylation remained unaffected in astrocytes (Figure 1C). Accordingly, phosphorylated tau was increased by about 40% in SGPL1-deficient neurons whereas no changes were detectable in astrocytes lacking SGPL1 when compared with the respective control cells (Figure 1C). This indicates that the increase in tau phosphorylation in hippocampus and cortex is due to hyperphosphorylation of tau in neurons.

### 3.2. Histone Acetylation Levels Vary in Different Cell Types Derived from Brains Lacking SGPL1

**Figure 2:**
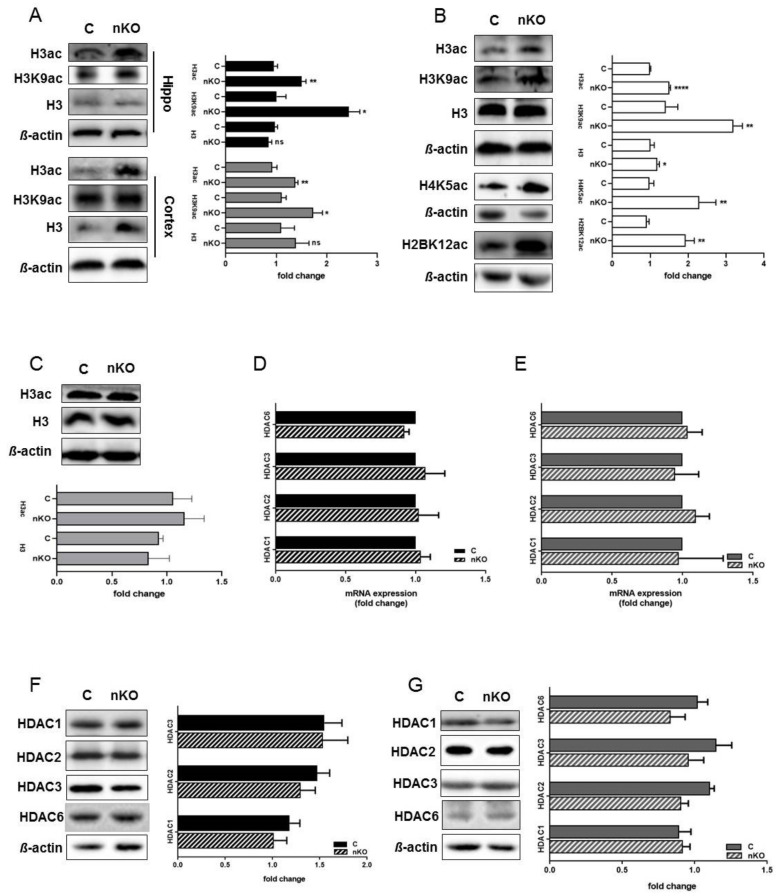
SGPL1 deficiency affects histone acetylation in the brain without affecting histone deacetylases (HDAC) expression. (**A**) Protein quantification of H3 pan-acetylation (H3ac), H3K9 acetylation (H3K9ac), and total H3 in the hippocampus (Hippo, black) and cortex (dark grey) from control (C) and SGPL1^fl/fl/Nes^ (nKO) mice. (**B**) Protein quantification of H3 pan-acetylation (H3ac), H3K9 acetylation (H3K9ac), total H3, H4K5 acetylation (H4K5ac), and H2BK12 acetylation (H2BK12ac) in astrocytes from control (C) and SGPL1^fl/fl/Nes^ (nKO) mice. (**C**) Protein quantification of H3 pan-acetylation (H3ac) and total H3 in primary neuronal culture from control (C) and SGPL1^fl/fl/Nes^ (nKO) mice. (**D**–**E**) qRT-PCR of HDAC1, 2, 3, and 6 in the hippocampus (**D**) and cortex (**E**) of control (C) and SGPL1^fl/fl/Nes^ (nKO) mice. (**F**–**G**) Protein quantification of HDAC1, 2, 3, and 6 in the hippocampus (**F**) and cortex (**G**) of control (C) and SGPL1^fl/fl/Nes^ (nKO) mice. For all: Bars mean +/− S.E.M, Student’s *t*-test with false discovery rate (FDR) correction, n = 3−7, * *q* < 0.05, ** *q* < 0.01, *** *q* < 0.001, ns = not significant.

Based on reports that tau stimulates chromatin relaxation [20] and that S1P accumulation elevates histone acetylation in tumorigenic cells [24], we analyzed histone acetylation in hippocampal and cortical slices from SGPL1^fl/fl/Nes^mice. We found that, upon S1P accumulation in the brain, acetylation of H3 is also significantly increased in both hippocampal and cortical slices by about 36% and 32%, respectively (Figure 2A). An investigation of specific acetylation sites revealed that K9 of H3 was significantly increased by about 45% in the hippocampus, and to a lesser extent (about 35%) in the cortex, as compared to the respective controls (Figure 2A). Interestingly, acetylation of all other lysine residues examined, including K14 and K18 was not affected by SGPL1 deficiency (Supplementary Figure S2). Next, we investigated acetylation of H3 in primary cultured cells derived from SGPL1^fl/fl/Nes^mice. We found that acetylation of H3 was significantly increased in astrocytes generated from SGPL1^fl/fl/Nes^mice (Figure 2B), whereas no changes of H3 acetylation were observed in primary cultured neurons of these mice (Figure 2C). Notably, the acetylation of H3 (H3ac) largely resembled that of lysine 9 of H3 (H3K9ac), whereas the total H3 level was only slightly increased (Figure 2B). These results strongly suggest that the increases in histone acetylation observed in the brain upon SGPL1 deficiency is due to epigenetic changes in astrocytes, rather than neurons. Interestingly, we also found that acetylation of H4 at K5 was significantly increased in astrocytes of SGPL1^fl/fl/Nes^mice, similar to what was shown previously in breast cancer cells (Figure 2B) [24]. Furthermore, we observed an increase in acetylation of H2B at K12, also consistent with a report of S1P accumulation in breast cancer cells (Figure 2B). Given the differences in histone acetylation observed, and given that S1P can act as an inhibitor of histone deacetylases [24], we next investigated whether the expression of histone deacetylases was affected in SGPL1^fl/fl/Nes^mice. However, we found that both mRNA and protein levels of HDAC1, 2, 3 and 6 remained unaffected in SGPL1^fl/fl/Nes^mice, compared to controls (Figure 2D–G). These results show that S1P accumulation in the brain has a cell type-specific effect on protein posttranslational modifications, without affecting the expression levels of the deacetylases responsible.

Together, these results indicate that different cell types can be responsible for interrelated effects detected when studying certain brain regions.

### 3.3. Calcium Chelation Reverses Both Tau Phosphorylation and Histone Acetylation in the Brain of SGPL1^fl/fl/Nes^mice

**Figure 3:**
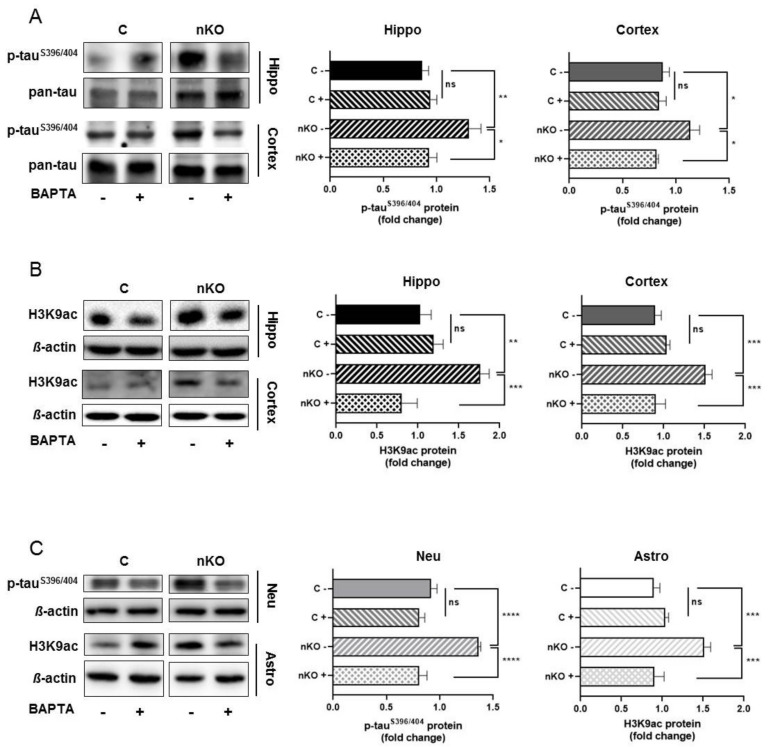
BAPTA-AM treatment reversed tau^S396/404^hyperphosphorylation and H3K9 acetylation in the brain of SGPL1^fl/fl/Nes^mice.(**A**) Protein quantification of phospho-tau^S396/404^ and total tau (pan-tau) in the hippocampus (Hippo, black) and cortex (dark grey) of control (**C**) and SGPL1^fl/fl/Nes^(nKO) mice with (+) and without (-) BAPTA-AM treatment. (**B**) Protein quantification of H3K9 acetylation (H3K9ac) and total H3 in the hippocampus (Hippo, black) and cortex (dark grey) of control (**C**) and SGPL1^fl/fl/Nes^ (nKO) mice with (+) and without (-) BAPTA-AM treatment. (**C**) Protein quantification of phospho-tau^S396/404^ and H3K9 acetylation (H3K9ac) in neurons (Neu, light grey) and astrocytes (Astro, white) in control (**C**) and SGPL1^fl/fl/Nes^ (nKO) mice with (+) and without (-) BAPTA-AM treatment. For all: Bars mean +/− S.E.M, One-Way ANOVA with Tukey’s post-hoc correction, n = 3−7, * *p* < 0.05, ** *p* < 0.01, *** *p* < 0.001, **** *p* < 0.0001, ns = not significant.

We have previously suggested that increased calcium concentrations might account for the neurotoxic effect of S1P in SGPL1-deficient neurons [14]. Calcium measurements in hippocampal slices of SGPL1^fl/fl/Nes^mice revealed a persistent elevation of basal calcium concentration in pyramidal neurons of the CA1 region amounting about 223 nM, a value that exceeds control concentrations by about 2.5-fold [27]. To find out whether elevated basal calcium concentration is linked to tau phosphorylation, we subjected hippocampal as well as cortical slices to BAPTA-AM treatment. Notably, calcium chelation by BAPTA reversed tau phosphorylation in SGPL1-deficient hippocampal and cortical slices to control values, specifically at the pathological phosphoepitope at serine residue S396/404 (Figure 3A), while phosphorylation of serine residues S262/356 was not affected by BAPTA (Supplementary Figure S3). Furthermore, we found that histone acetylation in the same samples also returned to control levels following BAPTA-AM treatment (Figure 3B).

### 3.4. Neuronal Tau Pathology and Augmented Histone Acetylation in Astrocytes of SGPL1^fl/fl/Nes^ Mice Are Calcium Dependent

As shown above, tau hyperphosphorylation appeared to be neuron specific, whereas the unusual increase of histone acetylation was restricted to astrocytes. The fact that, in hippocampal and cortical slices, BAPTA-AM reversed both parameters to control values prompted us to investigate the effect of calcium chelation in primary cultured neurons and astrocytes, respectively. Treatment of primary cultured neurons from SGPL1^fl/fl/Nes^mice with BAPTA-AM recapitulated the effect on tau phosphorylation described above in hippocampal and in cortical slices. Thus, the expression of phosphorylated tau at serineS396/404 returned to control values following calcium chelation (Figure 3C). Likewise, the treatment of primary cultured astrocytes derived from SGPL1^fl/fl/Nes^mice reversed the acetylation of H3 at K9 (H3K9ac) to control amounts (Figure 3C).

## 4. Discussion

SGPL1 deficiency in brain has been shown to affect neuronal health and to cause neuroinflammation accompanied by impairment of cognitive and motor skills in mice [17,18,28]. The central aim of the present study was to further unravel the molecular bases of the neurological phenotype of SGPL1^fl/fl/Nes^mice. We therefore investigated tau expression and phosphorylation in SGPL1-deficient animals. While the amount of total tau remained unaffected in the brains of SGPL1^fl/fl/Nes^mice, phosphorylation of tau at disease-relevant sites was significantly increased in brain slices and neuronal cultures derived from these mice. Based on reports from the early nineties that altered calcium homeostasis may be a key event leading to altered tau disposition and neuronal degeneration [29], we assumed that increase of phosphorylated tau in SGPL1-deficient brains could be linked to the increase of neuronal calcium in SGPL1^fl/fl/Nes^mice. Our results confirmed that at least one of the investigated disease-relevant phosphorylation sites can be reversed by BAPTA-AM treatment, indicating that the effect of SPGL1 deficiency on tau phosphorylation is also calcium-dependent. Although several changes of tau expression, mutation and posttranslational modifications were described in diverse pathologies of the central nervous system, hyperphosphorylation appears to be of particular importance for its pathologic function [19,30]. Our results show that SPGL1 deficiency in the brain can lead to a neuronal-specific, calcium-dependent hyperphosphorylation of tau at a site relevant to tauopathies. 

We have previously shown that SPGL1 deficiency leads to an accumulation of aggregate-prone proteins in the brain, along with deficits in motor coordination, learning and memory [17,18]. Furthermore, we have shown that SGPL1 deficiency on the one hand triggers the ubiquitin-proteasome system (UPS) in brains of SGPL1^fl/fl/Nes^mice [17], while on the other hand, it blocks neuronal autophagy flux at an early stage [18]. The implication of both systems in neuronal health and death is well established [31,32,33,34]. Notably, both systems are also responsible for tau clearance [35]. While numerous reports suggested that tau is a proteasomal substrate, other studies found that the autophagy/lysosomal pathway is the primary degradation machinery for tau [35]. Our results regarding unchanged amounts of total tau could be explained by these opposing effects of SGPL1 deficiency on the two degradation systems in neurons [17,18]. Further studies into the molecular mechanisms by which SGPL1 deficiency and/or S1P accumulation in the brain affect tau hyperphosphorylation, clearance and pathology could therefore be of great interest to the field of tauopathies.

Although tau is predominantly produced by neurons in the brain, tau pathology is not restricted to neurons [30,36]. However, astrocytes derived from SGPL1-deficient brains did not show any changes in tau expression or phosphorylation. One explanation might be the fact that many studies regarding tau pathology were performed in transgenic animals harboring the wild-type or mutant human tau transgene [30,37]. On the other hand, apart from hyperphosphorylation, microglial activation and thus neuroinflammation appear to be essential for astrocytic tau pathology [30]. We have recently shown that in SGPL1^fl/fl/Nes^mice, microglial activation and hence release of pro-inflammatory cytokines including interleukin (IL) 6 (IL-6) and tumor necrosis factor alpha (TNF-α), is caused by S1P released from SGPL1-deficient astrocytes [28]. Whether and how these factors are involved in the tau hyperphosphorylation of SGPL1-deficient astrocytes has to be clarified in future studies.

It is well-established that mobilization of intracellular calcium stores is a universal signaling mechanism that cells employ for responding to a wide range of external stimuli [38]. Notably in SGPL1-deficient neurons, this stimulus is S1P [5]. We also found it rather interesting that both S1P [24] and tau, downstream of its pathologic accumulation [22] have been shown to affect histone acetylation, though in different cellular contexts. Specifically, S1P produced by overexpression of SK2 in the nucleus of breast cancer cells was shown to inhibit HDAC 1 and 2, thus increasing acetylation of H3 at K9, of H4 at K5, and rather weakly that of H2B at K12 [24]. Moreover, a compromised acetylation homeostasis has been suggested to be intimately coupled with neurodegeneration [39]. We therefore decided to investigate whether SGPL1 deficiency in the brain could exert an analogous effect. Intriguingly, we also found calcium-dependent increases in H3K9 and H2BK12 acetylation in brain slices and primary cultured astrocytes, but not in neurons derived from SGPL1^fl/fl/Nes^mice, without affecting the overall expression levels of HDAC1, 2, 3, or 6. These results indicate that SGPL1 deficiency also plays a role in histone posttranslational modifications in astrocytes, though further studies are needed to elucidate the role of this epigenetic disruption on overall brain health.

In SGPL1-deficient mouse embryonic fibroblasts (MEFs) an increase of acetylated H3K9 was reported, but no changes in the level of H4 and H2B acetylation could be detected [40]. Despite this similarity, the underlying molecular mechanisms appear to diverge in the three cell types. In the SK2-overexpressing cancer cell lines, nuclear S1P was shown to directly interact and thus inhibit HDACs1 and 2 [24] while in SGPL1-deficient MEFs a reduced expression of HDACs1, 2, and 3 was reported [40]. Intriguingly, the decreased expression of HDACs was correlated with an elevated basal calcium level in SGPL1-deficient MEFs [40]. By contrast, in hippocampal and cortical slices derived from SGPL1-deficient murine brains, no changes in the expression of HDACs could be observed. However, calcium chelation by BAPTA-AM restored histone acetylation, suggesting that calcium mediates the effect of S1P on histone acetylation independent of HDAC expression. Given these differences between models, we cannot make assumptions as to the specific molecular mechanism responsible for altered histone acetylation in our system.

Interestingly, epigenetic dysregulation currently attracts much attention as a pivotal player in aging and age-related neurodegenerative disorders, such as AD, Parkinson’s disease and Huntington’s disease, where it may mediate interactions between genetic and environmental risk factors, or directly interact with disease-specific pathological factors [23,41]. Furthermore, a recent epigenome-wide association study using H3K9ac as a marker for transcriptionally active open chromatin revealed that tau pathology is associated with broad changes in the brain’s epigenome [22]. This study was conducted in aged human dorsolateral prefrontal cortices. Also, in post-mortem human brains from AD patients, tau pathology was correlated with augmented H3 and H4 acetylation [23]. Given our findings that SPGL1 deficiency results in both altered tau homeostasis and histone acetylation, further studies into how SPGL1 deficiency and/or S1P accumulation fit into these complex systems are needed. While a closer look in primary cultured neurons and astrocytes uncovered a cell type specificity to the effect of SGPL1 deficiency, this does not exclude their association in the nervous tissue. At present, the association of tau pathology and histone acetylation appears rather conflicting and far from clear. It has been reported that specific inhibition of HDAC3 and consequently an increased acetylation of H3 and H4 was shown to reduce tau phosphorylation at disease- associated sites, including serine 396, and was proposed as a novel neuroprotective mechanism [42]. Similarly, the specific inhibition of HDAC6 caused a significant reduction of tau phosphorylation [43]. HDAC6 inhibition also increased acetylation of Hsp90 which caused ubiquitination of phosphorylated tau thus alleviating abnormal tau accumulation [43]. Despite many open questions, we feel that our results show that SGPL1 deficiency impacts brain health and might help explain the potential molecular mechanisms underlying the diverse neuropathological phenotypes in humans harboring mutations in the *SGPL1* gene [8].

## 5. Conclusions

Our results indicate that SGPL1 depletion augments tau phosphorylation in neurons and simultaneously enhances histone acetylation in astrocytes suggesting a negative impact on brain health. On the other hand, immunohistochemical analysis in frontal and entorhinal cortices from 56 human AD brains revealed an augmented SGPL1 expression correlating with amyloid deposits [12]. The same study also reported a decreased expression of sphingosine kinase 1 as well as of S1PR1 suggesting a global deregulation of S1P signaling in human AD brains [12]. Our results rely primarily on SGPL1 deficiency. Hence, although sites relevant to tauopathies including AD are hyperphosphorylated, we feel that, due to the multitude of phosphorylation sites in tau and the complexity of this phenomenon [44], more studies are needed to finally understand the function of SGPL1 in tauopathies. At present, our findings are first and foremost interesting for a better understanding of the phenotype of humans with insufficient SGPL1 activity [8]. 

## Supplementary Materials

The following are available online at https://www.mdpi.com/2073-4409/9/10/2189/s1, Figure S1: Neural-specific depletion of SGPL1; Figure S2: SGPL1 deficiency has no effect on acetylaztion of Histone3 at lysine 14 and 18; Figure S3: BAPTA-AM treatment has no effect on tau^S262/356^ hyperphosphorylation in the brain of SGPL1^fl/fl/Nes^ mice.
